# Responding to Sexual Objectification: The Role of Emotions in Influencing Willingness to Undertake Different Types of Action

**DOI:** 10.1007/s11199-018-0912-x

**Published:** 2018-04-09

**Authors:** Lee Shepherd

**Affiliations:** Department of Psychology, Northumbria University, Northumberland Building, Northumberland Road, Newcastle upon Tyne, NE1 8ST UK

**Keywords:** Emotions, Objectification, Sexual harassment, Action tendency, Sexism

## Abstract

Women may respond to being sexual objectified in different ways, such as confronting the perpetrator, ignoring the action, blaming oneself or considering the action as flattering. However, there has been little research looking at what predicts each of these different responses. The present research assessed the role of emotions in promoting and deterring different anticipated responses to sexual objectification. In both Studies 1 (*n* = 189) and 2 (*n* = 187), female participants were asked to imagine themselves in a situation where they received an inappropriate sexual comment. Participants then rated the emotions they believed they would feel and how they thought they would act in the situation. I found that expecting feelings of anger resulted in women thinking they would confront the perpetrator (i.e., undertake an active response) and that expecting disgust negatively predicted the belief that they would view the action as flattering (i.e., anticipate a benign response). By contrast, expecting shame resulted in women believing they would blame themselves (i.e., undertake a self-blame response). Study 2 also found that expecting pride was positively associated with anticipating a benign response. These findings suggest that the emotions that women expect to feel influence their anticipated responses to sexual objectification. It is, therefore, important to consider the emotional reactions that women have to instances of sexual objectification.

**Electronic supplementary material:**

The online version of this article (10.1007/s11199-018-0912-x) contains supplementary material, which is available to authorized users.

Many countries have celebrated, or are preparing to celebrate, important anniversaries for women’s rights. For example, in 2018 the United Kingdom celebrated 100 years since women were given the right to vote. Similarly, the United States will be celebrating this anniversary in the next few years. However, despite such landmark victories for women’s rights occurring nearly a century ago, women are still subjected to various sexist actions in their everyday lives (Becker and Swim 2011; Swim et al. 2001). Women may respond to such actions in different ways (Swim and Hyers 1999). For example, women may take an *active* response and confront the perpetrator (Wang and Dovidio 2017). By contrast, targets may undertake a *passive* response and ignore the action (Fairchild and Rudman 2008). Alternatively, targets may *self-blame* and think that they brought the action upon themselves (Schneider et al. 2001). For example, targets of sexual objectification may think that they have brought it upon themselves through the way they have dressed. Finally, they may take a *benign* response, believing that the perpetrator meant well by the action. For example, the action could be viewed as a joke (Mallett et al. 2016). Alternatively, women may view sexual objectification as a compliment (Sáez et al. 2016). The aim of this research was to assess the factors that predict the type of anticipated response that is likely to be undertaken following instances of sexism.

A variety of factors have been shown to influence how women respond to sexism and discrimination (for a discussion, see Becker et al. 2014). Indeed, the willingness to take action varies depending on the type of sexist action (Ayres et al. 2009), the target’s exposure to hostile and benevolent sexism (Becker and Wright 2011), the target’s gender identification (Wang and Dovidio 2017), the power of the perpetrator (Ashburn-Nardo et al. 2014) and the expected social costs of confronting the perpetrator (Shelton and Stewart 2004). Although such factors are influential, it is also important to consider the role of emotions. This is especially important given that a variety of emotions may be felt after being exposed to sexism (Chaudoir and Quinn 2010; Guizzo et al. 2017). However, there has been relatively little research assessing whether emotions influence the likelihood of an active, passive, self-blame or benign response being anticipated. This is especially true for sexism that involves sexual objectification. Therefore, the aim of the present research is to assess the role of expected emotions in predicting how women believe they would respond to instances of sexual objectification.

## Emotions and Sexual Objectification

Sexual objectification may be defined as someone being regarded as an object for sexual pleasure rather than as a human being. This may involve body evaluation (e.g., inappropriate sexual remarks or leering) or unwanted sexual advances (e.g., being groped; Kozee et al. 2007). Such sexist experiences may elicit a variety of emotions (Chaudoir and Quinn 2010). For example, research has suggested that being objectified is likely to promote feelings of anger (Roosmalen and McDaniel 1999) and disgust (Fredrickson et al. 1998). The emotion that is elicited is likely to depend on the individual’s interpretation of the situation (Smith and Lazarus 1993). Anger is likely when a moral violation is viewed as harming the target’s individual rights and disgust is likely when this action is believed to harm the purity of the body or soul (Rozin et al. 1999).

Sexual objectification may also elicit self-critical emotions (Van Vliet 2009). There are various self-critical emotions (Gausel and Leach 2011). For example, following an instance of sexual objectification, people may feel shame when they think this action reflects a specific self-defect (e.g., “Being objectified undermines my competence”), inferiority when this reflects a global self-defect (e.g. “Being objectified suggests I am powerless and worthless”) and rejection when they believe that they are likely to be judged negatively by others (e.g., “Being objectified might result in others not respecting me”). Importantly, although researchers often focus on the elicitation of image-threatening emotions in perpetrators of transgressions, targets may also feel these emotions when they believe that the harmful action is detrimental to their image (Van Vliet 2009). Targets are likely to feel such emotions because the action undermines their status (Matheson and Anisman 2009). For example, objectification undermines the target’s moral and competent status (Heflick et al. 2011), potentially damaging the target’s image and thus resulting in self-critical emotions.

It is also important to discuss positive emotions that may be felt after being subjected to a sexually objectifying behaviour. Given the mass of research demonstrating the negative effects of sexual objectification (Calogero 2004; Calogero and Thompson 2009; Fredrickson and Roberts 1997; Noll and Fredrickson 1998; Rosenthal et al. 2016), discussing positive emotions may be regarded as counter-intuitive. However, appearance-related compliments may promote positive feelings (Herbozo and Thompson 2006). Similarly, sexual objectification may elicit a benign response, such as considering the action as flattering (Fairchild and Rudman 2008; Roosmalen and McDaniel 1999) or even enjoyable (Sáez et al. 2016). Therefore, although such responses may be less frequent than active or passive responses (Fairchild 2007), it is still important to explore their antecedents. Pride is felt when an individual views one of their actions or attributes positively (Tracy and Robins 2007). As such, pride may be felt if someone believes that they were subjected to the sexually objectifying behaviour because of their attractiveness.

## Responses to Objectification

Emotions are associated with a variety of action tendencies (Frijda et al. 1989). Some emotions are likely to promote confrontation, whereas others may cause avoidance (Roseman et al. 1994). Therefore, the emotions that are experienced following sexual objectification may influence the response that is subsequently undertaken. Although anger and disgust are distinct emotions, they both are likely to stem from moral violations (Rozin et al. 1999). Therefore, researchers have argued that both these emotions form the basis of moral outrage (Darley and Pittman 2003; Salerno and Peter-Hagene 2013). Moral outrage has been associated with the desire to take action and punish the perpetrator (Bastian et al. 2013; Montada and Schneider 1989). In line with this reasoning, research has suggested that feelings of anger motivate people to take proactive responses against transgressions (van van Zomeren et al. 2012). Indeed, this process has been demonstrated when people observe others being objectified (Chaudoir and Quinn 2010), such as watching women being objectified on television (Guizzo et al. 2017). Therefore, these emotions based in moral outrage are likely to promote active responses to sexual objectification and thus reduce passive responses.

Shame and inferiority, on the other hand, stem from the belief that the transgression occurred because of a self-defect (Gausel and Leach 2011). This internalisation process may result in these emotions promoting self-blame. Moreover, although shame has been traditionally associated with withdrawal (Tangney et al. 2007), more recent research suggests that it is feelings of rejection that promote withdrawal rather than shame (Gausel et al. 2012; Gausel et al. 2016). This reasoning suggests that feeling rejection following sexual objectification is likely to deter active and promote passive responses, such as ignoring the action.

To my knowledge, there has been little research assessing the role of pride on women’s responses to sexual objectification. However, research has suggested that women who enjoy sexualisation are likely to hold traditional gender attitudes and believe that gender relations are fair (Erchull and Liss 2013; Liss et al. 2011). This suggests that gaining pleasure from objectification (i.e., feeling pride) may result in women being less likely to view such actions as sexist. Based on this speculation, I hypothesised that feeling proud of receiving sexually objectifying behaviours is likely to promote benign responses, such as considering the action as flattering.

## The Present Studies

The theoretical account I described suggests that there are a variety of emotions that may be elicited following instances of sexual objectification and that these emotions are likely to influence women’s willingness to undertake a particular response. However, research has generally focused on the role of anger in promoting active responses to sexism (Chaudoir and Quinn 2010; Guizzo et al. 2017). By contrast, there has been little research assessing the roles of disgust, self-critical emotions, and pride in influencing the type of responses undertaken following sexual objectification. Given the strong theoretical account I described, it is important to go beyond the focus of anger to confrontation and assess the role of other emotions in differentiating the type of response that is elicited following sexual objectification. Therefore, the aim of my research was to assess the role of different expected emotions in influencing how women think they are likely to response to instances of sexual objectification. Although these patterns ideally would be explored by examining women’s reactions to actually being objectified, a first step in doing such research is to explore women’s expected emotions and anticipated responses to imagined objectification. The latter is the focus of the present research.

This was tested using both correlational (Study 1) and experimental studies (Study 2). These two studies assessed numerous research questions. First, I tested whether expecting to feel moral outrage-based emotions (i.e., anger and disgust) positively predicts women’s belief that they would undertake active responses. Second, I tested whether expecting to feel shame and inferiority increases women’s belief that self-blame would be undertaken. Third, I assessed whether expecting to feel rejection negatively predicts women’s belief that active responses would be undertaken and positively predicts the belief that passive responses would be undertaken. In addition, Study 2 tested whether expecting pride positively predicts the belief that benign responses would be elicited.

## Study 1

Study 1 assessed the role of the expected negative emotions in influencing the anticipated responses to sexual objectification. In line with previous research (Teng et al. 2015), female participants were asked to imagine themselves in a situation where they received an inappropriate sexual remark from a male stranger. Participants then rated their expected emotions toward receiving this comment and their anticipated responses to sexual objectification. As I noted previously, given my use of vignettes, my study assessed each participant’s anticipated response rather than their actual emotions and responses. Moreover, because previous research has suggested that sexual objectification increases body shame and surveillance (Fairchild and Rudman 2008), it was important to check that any association between the expected emotions and the anticipated responses was not due to body shame or surveillance. As such, these subscales were included in my analyses as covariates. Finally, age was included as a covariate to assess whether this influenced perceptions of objectification.

### Method

#### Participants and Design

Participants were recruited for this online study through adverts on social media and recruitment websites. The criteria for taking part was that the participant had to view themselves as female and be 18 years or older. For ethical reasons participants were told not to take part if they had been diagnosed with an eating disorder or were likely to feel distressed when discussing sexual objectification. A total of 301 participants started this survey. There were 109 participants removed for not reaching the end of the survey and three who were removed because they were under 18 years old. Therefore, the final sample consisted of 189 women, aged 18–69 years (*M =* 25.79, *SD* = 7.49). Participants were more likely to state they were in a relationship (*n* = 105, 55.56%) rather than single (*n* = 77, 40.74%), divorced/separated (*n* = 3, 1.59%), widowed (*n* = 1, .53%) or select “other” (*n* = 3, 1.59%). When asked to state their nationality, participants were more likely to state a country in North America (*n* = 126, 66.67%) than Europe (*n* = 21, 11.11%), Australia or New Zealand (*n* = 16, 8.47%), Asia (*n* = 7, 3.70%) or South America (*n* = 1, .53%). Five participants stated they were of mixed nationality (2.65%) and 13 (6.88%) did not provide sufficient information to determine their nationality.

This online study used a correlational design. The predictor variables were their expected emotions (anger, disgust, shame, inferiority, and rejection). The outcome variables were their anticipated responses to sexual objectification (active, passive, self-blame, and benign). Measures of body surveillance and body shame were also included. These variables were entered into the analysis as covariates. Finally, age was included into the analysis as a covariate.

#### Materials and Procedure

Ethical approval for this study was granted through the author’s institutional review board. Participants were first asked to read an information sheet describing the study. Once the participant had given consent, they were asked to complete the demographic measures. Next, a vignette was used to induce sexual objectification. Participants were asked to imagine that they were at their local gym and that during their exercise session they were engaging in a polite conversation with a man. To induce sexual objectification, participants were asked to imagine that during the conversation this man said: “You were looking good on the treadmill. It seems to be working for you. You have a great body and an amazing ass.” (The full vignette is available in an online supplement.)

Next, participants rated how they were likely to feel in this situation. Participants were asked to rate the extent to which “The way that I was treated in this situation would make me feel [emotion word],” using a scale from 1 (*Not at all*) to 5 (*Very much*). Based on previous research (Livingstone et al. 2011; Shepherd et al. 2013), the anger words were angry, annoyed, and furious (α = .90). The disgust words were disgust and repulsed (*r* = .89, *p* < .001). The shame (ashamed, disgraced, and humiliated; α = .82), inferiority (inferior and vulnerable; *r* = .56, *p* < .001), and rejection (rejected, withdrawn, and alone; α = .71) words were taken from previous research (Gausel et al. 2012). All items defining each emotion were averaged so that higher scores indicate expectations of stronger emotionality.

Participants then rated their anticipated responses to sexual objectification, using a scale based on Fairchild and Rudman (2008; the full scale is available in the online supplement). Participants were asked: “How likely are you to…,” using a scale from 1 (*Not at all*) to 5 (*Extremely likely*). There were four active response items (e.g., “Let him know you do not like what he is doing”; α = .77), seven passive response items (e.g., “Ignore the whole thing”; α = .89), four self-blame items (e.g., “Blame yourself for what happened”; α = .81), and five benign response items (e.g., “Consider it flattering”; α = .80; for a full scale, see Fairchild and Rudman 2008).

Finally, participants completed the surveillance and body shame subscales from the Objectified Body Consciousness Scale (for full scales, see McKinley and Hyde 1996). There were eight items measuring surveillance (e.g., “I rarely think about how I look” [reverse scored]; α = .87) and an additional eight items measuring body shame (“I feel ashamed of myself when I haven’t made the effort to look my best”; α = .84). Each item was rated on a 7-point Likert scale from 1 (*Strongly disagree*) to 7 (*Strongly agree*). Items were averaged within each scale so that higher scores capture greater surveillance or body shame.

#### Statistical Analysis

Initially, correlation analyses were conducted to assess the association between the expected emotions and anticipated responses. Following this, confirmatory factor analysis was performed to ensure that the emotions were separate constructs. Multiple regression analyses were then used to assess the unique predictive power of the expected emotions on the different anticipated responses. A separate regression analysis was conducted for each of the four anticipated responses. Given that the analyses was repeated numerous times, Bonferroni corrections were used to control for the family-wise error rate. Therefore, *p*-values had to be below .0125 (i.e., .05/4) for the result to be regarded as significant.

### Results

During the data sorting process, it became apparent that there were some outliers for the anticipated self-blame response and age variables (i.e., scores ±3 standard deviations from the mean). Therefore, logarithmic and inverse transformations were performed on these variables, respectively, to correct for outliers.

#### Associations between Variables

Correlation analyses indicated that expected anger, disgust, shame, and inferiority were positively associated with anticipating an active response (see Table 1). By contrast, expected anger and disgust were negatively associated with anticipating a passive response. There was a significant positive association of expected shame, inferiority, and rejection with anticipating a self-blame response. Finally, the negative expected emotions were negatively associated with anticipating a benign response. Surveillance was negatively associated with expected anger and anticipating an active response. Body shame was positively associated with expected shame, inferiority, rejection, and anticipating a passive and self-blame response. Age was positively associated with expected anger. There were some close associations between the expected emotions. Indeed, expected anger and disgust were closely associated. Moreover, expected shame and inferiority were closely associated. Therefore, it was important to assess whether these were separate constructs, as suggested by previous research (Gausel and Leach 2011; Salerno and Peter-Hagene 2013).

**Table 1:** Descriptive statistics and correlations between the expected emotions and anticipated responses, Study 1

		Correlations										
	*M* (*SD*)	1	2	3	4	5	6	7	8	9	10	11
Expected emotions												
1. Anger	2.87 (1.21)	–										
2. Disgust	3.39 (1.43)	.68***	–									
3. Shame	2.19 (1.10)	.53***	.47***	–								
4. Inferiority	2.69 (1.20)	.52***	.48***	.65***	–							
5. Rejection	1.91 (.92)	.52***	.49***	.60***	.60***	–						
Anticipated responses												
6. Active response	2.54 (.98)	.47***	.50***	.20**	.17*	.13	–					
7. Passive response	2.78 (1.00)	−.31***	−.33***	−.12	−.05	−.06	−.58***	–				
8. Self-blame response	.21 (.19)	.13	.07	.42***	.36***	.33***	−.14	.25**	–			
9. Benign response	1.95 (.81)	−.58***	−.62***	−.40***	−.44***	−.33***	−.31***	.41***	−.02	–		
Covariates												
10. Surveillance	4.55 (1.20)	−.15*	−.13	−.05	−.02	−.12	−.17*	.09	.12	.13	–	
11. Body shame	3.42 (1.30)	.05	.07	.23**	.23**	.16*	−.10	.18*	.32***	−.01	.54***	–
12. Age	1.02 (.01)	.17*	.01	.02	−.01	.10	.06	.03	.01	−.04	−.14	−.09

*p < .05. **p < .01. ***p < .001

#### Confirmatory Factor Analysis

Confirmatory factor analysis was used to assess the structure of the expected emotion constructs. This was conducted in AMOS Version 22 (Arbuckle 2013). The model was based on maximum likelihood estimation. In the hypothesised model, the five expected emotions were separate constructs. This model fit the data well: χ^2^(55) = 107.43, *p* < .001, comparative fit index (CFI) = .97, normed fit index (NFI) = .93 and root mean squared error of approximation (RMSEA) = .07. This was contrasted against an alternative four-factor model in which expected anger and disgust were combined. This alternative model did not fit the data well: χ^2^(59) = 294.59, *p* < .001, CFI = .85, NFI = .82 and RMSEA = .15. Moreover, the hypothesised model fit the data significantly better than this alternative model: *∆χ*^*2*^(4) = 187.16, *p* < .001.

The hypothesised model was also contrasted against a three-factor model in which expected anger and disgust were separate constructs, but the self-critical emotions were combined into a single construct. This model adequately fit the data: χ^2^(62) = 160.19, *p* < .001, CFI = .94, NFI = .90 and RMSEA = .09. However, the hypothesised model fit the data significantly better than this alternative model: *∆χ*^*2*^(7) = 52.76, *p* < .001. This suggested that the expected emotions were five separate constructs. Importantly, further analysis demonstrated that including these five expected emotions into a regression analysis created a lowest tolerance value of .45. The fact that this lowest tolerance value was greater than .20 suggests it was unlikely that the results would be bias by multicollinearity (Menard 1995).

#### Role of Expected Emotions in Predicting the Anticipated Responses

Multiple regression analyses were conducted to assess the role of the expected emotions in predicting the different anticipated responses to sexually objectifying behaviours. In these analyses, the expected emotions were the predictor variables and the anticipated responses were the outcomes. Surveillance, body shame, and age were covariates. The predictors were entered into the model in Step 1 and the covariates were entered into the model in Step 2. This tested whether any associations between the expected emotions and anticipated responses remained after controlling for the covariates. Importantly, given the analysis was repeated four times (once for each anticipated response), Bonferroni corrections were applied to reduce family-wise error. As such, results were regarded were significant when the *p*-value was below .0125 (i.e., .05/4).

##### Anticipated Active Response

For active responses, Step 1 accounted for 32% of the variance, *F*(5, 182) = 16.81, *p* < .001. Anticipating an active response was positively predicted by expected anger and disgust (see Table 2). After making Bonferroni corrections, expected rejection did not significantly predict anticipating an active response. Expected shame and inferiority were non-significant predictors. Step 2 accounted for 33% of the variance in anticipating an active response, *F*(8, 179) = 10.89, *p* < .001. However, adding the covariates did not improve the predictive power of the model, ∆R^2^ = .01, *F*(3, 179) = 1.01, *p* = .392. None of the covariates were significant predictors. Importantly, the expected emotions remained significant predictors after accounting for the covariates. These results reflect the fact that expected anger and disgust positively predict the anticipation of undertaking an active response.

**Table 2:** Regression analysis assessing the role of the expected emotions in predicting anticipated responses, Study 1

	Active response		Passive response		Self-blame response		Benign response	
	Step 1 *B* (*SE*)	Step 2 *B* (*SE*)	Step 1 *B* (*SE*)	Step 2 *B* (*SE*)	Step 1 *B* (*SE*)	Step 2 *B* (*SE*)	Step 1 *B* (*SE*)	Step 2 *B* (*SE*)
Expected emotions								
Anger	.28*** (.07)	.27*** (.07)	−.22** (.08)	−.22** (.08)	−.02 (.02)	−.01 (.02)	−.18** (.05)	−.17** (.05)
Disgust	.27*** (.06)	.27*** (.06)	−.21** (.07)	−.20** (.07)	−.03 (.01)	−.02 (.01)	−.24*** (.04)	−.24*** (.04)
Shame	.02 (.08)	.03 (.08)	−.03 (.09)	−.06 (.09)	.06*** (.02)	.05** (.02)	−.04 (.06)	−.04 (.06)
Inferiority	−.08 (.07)	−.07 (.07)	.12 (.08)	.11 (.08)	.03 (.02)	.03 (.02)	−.11 (.05)	−.11 (.05)
Rejection	−.22 (.09)	−.22 (.09)	.15 (.10)	.12 (.10)	.03 (.02)	.03 (.02)	.11 (.07)	.11 (.07)
Covariates								
Surveillance	–	−.04 (.06)	–	−.06 (.07)	–	.003 (.01)	–	.03 (.05)
Body shame	–	−.05 (.06)	–	.17* (.06)	–	.03 (.01)	–	.02 (.04)
Age	–	.61 (6.83)	–	8.99 (7.55)	–	.63 (1.37)	–	.52 (4.99)
R^2^	.32***	.33***	.18***	.21***	.24***	.28***	.47***	.48***
∆R^2^	–	.01	–	.04	–	.04	–	.004

*p < .0125. **p < .01. ***p < .001

##### Anticipated Passive Response

Step 1 explained 18% of anticipated passive response variance, *F*(5, 182) = 7.73, *p* < .001. This was due to the anticipation of undertaking a passive response being negatively associated with expected anger and disgust (see Table 2). By contrast, expected shame, inferiority, and rejection were non-significant predictors. Step 2 accounted for 21% of the variance, *F*(8, 179) = 6.03, *p* < .001. Adding the covariates did not significantly improve the model after accounting for Bonferroni corrections, ∆R^2^ = .04, *F*(3, 179) = 2.80, *p* = .042. Interestingly, body shame was positively associated with anticipating a passive response. Expected anger and disgust remained significant negative predictors. These results suggest that expected anger and disgust deter, whereas body shame promotes, the anticipation of undertaking a passive response.

##### Anticipated Self-Blame Response

Step 1 accounted for 24% of variance in anticipating a self-blame response, *F*(5, 182) = 11.29, *p* < .001. Anticipated self-blame was positively associated with expected shame (see Table 2). In Step 2, 28% of the variance was explained, *F*(8, 179) = 8.56, *p* < .001. However, after making Bonferroni corrections, adding the covariates did not significantly improved the model, ∆R^2^ = .04, *F*(3, 179) = 3.29, *p* = .022. Expected shame remained a significant predictor after accounting for the covariates. The covariates did not significantly predict self-blame. These results reflect the fact that expected shame seemed to promote the anticipation of undertaking a self-blame response.

##### Anticipated Benign Response

For benign responses, the emotions accounted for 47% of the variance, *F*(5, 182) = 32.70, *p* < .001 (see Step 1). This was due to expected anger and disgust negatively predicting the anticipation of undertaking a benign response (see Table 2). Although Step 2 accounted for 48% of the variance, *F*(8, 179) = 20.41, *p* < .001, this did not improve the predictive power of the model, ∆R^2^ = .004, *F*(3, 179) = .43, *p* = .731. This was due to the covariates being non-significant predictors. Importantly, including these covariates did not alter the predictive power of expected anger and disgust. These results suggest that expected anger and disgust reduce the anticipation of undertaking a benign response.

### Discussion

The aim of Study 1 was to assess the role of expected emotions in predicting different anticipated responses to sexual objectification. In line with the hypotheses, expecting feelings of anger and disgust positively predicted the anticipation of active responses, but were negatively associated with the anticipation of passive and benign responses. Similarly, expected shame was positively associated with the anticipation of a self-blame response. Overall, the present research shows the predictive role of expected emotions in influencing the responses that are anticipated following instances of sexual objectification.

Although these findings are interesting, it is also important to consider the limitations of Study 1. First, this study used a correlational design, preventing causality from being inferred. Second, it focused on the expected negative emotions associated with sexual objectification. Given that some participants had an anticipated benign response, it is also important to account for the role of positive emotions. This is especially important given that sexual objectification may be perceived as a positive action (Erchull and Liss 2013; Liss et al. 2011; Roosmalen and McDaniel 1999). These limitations were addressed in Study 2.

## Study 2

There were three main differences between Studies 1 and 2. First, Study 2 enhanced Study 1 by using an experimental approach. In line with previous research (Teng et al. 2015), sexual objectification was manipulated through the use of vignettes. Participants in the objectified (but not the control) condition were asked to imagine themselves in a situation where they received an inappropriate sexual comment. The use of an experimental study allowed for causality to be inferred. Moreover, the combination of this with indirect effect analysis allowed the researcher to test whether the effect of objectification on the anticipated responses occurred via the expected emotions. Second, whereas Study 1 focused on expected negative emotions, Study 2 included a measure of pride to assess whether projecting this positive emotion influences anticipated benign responses. Third, Study 1 demonstrated that the relationship between most of the expected emotions and the anticipated responses was unlikely to be due to surveillance or body shame. However, other covariates could account for the associations. For example, the responses have been associated with self-objectification (Fairchild and Rudman 2008). Therefore, self-objectification was measured as a covariate in Study 2.

### Method

#### Participants and Design

Study 2 was advertised to participants through social media and recruitment websites. To take part, participants had to view themselves as female and be 18 years or older. For ethical reasons, participants were asked not to take part in this study if they had been diagnosed with an eating disorder or were likely to feel distressed when discussing objectification. Initially, 306 participants started this study. However, 118 participants were removed for not completing the study. There was also one participant whose age score was an outlier, even after applying an inverse transformation. Therefore, this participants was removed from the data, leaving a sample of 187 women. The age range was 18–62 years (*M* = 25.65, *SD* = 6.68). Participants were more likely to state they were in a relationship (*n* = 99, 52.94%) than single (*n* = 81, 43.32%), divorced/separated (*n* = 1, .53%), widowed (*n* = 1, .53%) or select “other” (*n* = 4, 2.14%). When asked about their nationality, participants were more likely to state a nation that is part of North America (*n* = 114, 60.96%) than Europe (*n* = 38, 20.32%), Australia or New Zealand (*n* = 11, 5.88%), Asia (*n* = 5, 2.67%) or Africa (*n* = 1, .53%). Five participants stated they were of mixed nationality (2.67%) and 13 participants (6.95%) did not give sufficient information to determine their nationality.

Study 2 used a between-participants experimental design. The independent variable was sexual objectification (control versus objectified). In the control condition, participants read a vignette that did not include overt sexual objectification, whereas in the objectified condition the vignette stated they had received an inappropriate sexual comment. The dependent variables were the participant’s anticipated response to the action (active, passive, self-blame or benign). The mediating variables were the expected emotions. Self-objectification and age were covariates.

#### Materials and Procedure

The author’s institutional review board provided ethical approval for this study. After giving consent, participants provided demographic information. This was followed by the sexual objectification manipulation. Participants in both conditions were asked to imagine themselves exercising at their local gym and that during their exercise session they had a conversation with a man they had never met before. Participants in the control condition were asked to imagine that during this conversation the man said: “You were looking good on the treadmill. Your exercise regime seems to be working for you.” As such, these participants received an ambiguous comment. By contrast, participants in the objectified condition were asked to imagine the man said: “You were looking good on the treadmill. Your exercise regime seems to be working for you. You have a great body and an amazing ass.” Therefore, these participants were asked to imagine they received an overt sexually objectifying comment. (The full vignettes are available in the online supplement.)

Participants then completed the measures. First, participants rated their expected feelings in this situation. Participants were asked to rate whether “Being treated in this way would make me feel [emotion word],” using a scale from 1 (*Not at all*) to 5 (*Very much*). The words used in the scales for expected anger (α = .90), shame (α = .82), inferiority (*r =* .54, *p* < .001), rejection (α = .65) and disgust (*r* = .84, *p* < .001) were identical to Study 1. The pride words were proud, satisfied, and feel good about myself (α = .91). The researcher was concerned that the inclusion of numerous aversive emotions may lead participants in the control condition to believe that there may be another condition containing a more overt form of sexual objectification. Therefore, to prevent this possibility, indifference was also measured. The emotion words for this measure were indifferent, apathetic, and ‘unconcerned’ (α = .57). Although the scale was unreliable, this was not a concern because this scale was only included to disguise the presence of a manipulation and was therefore not entered into the analysis. This was followed by the anticipated response scales. These scales were identical to Study 1. Importantly, the active (α = .72), passive (α = .85), self-blame (α = .84), and benign (α = .67) response scales had adequate reliability. In Study 1, the order of the expected emotion and anticipated response items was not randomised. Because of this, it could be argued that the order of the items could have biased the results. Therefore, in Study 2 the order of the expected emotion and anticipated response items was randomised to ensure that this was not the case.

Finally, participants completed the self-objectification measure. Similar to previous research (Noll and Fredrickson 1998), participants were presented with six observable (physical attractiveness, coloring, weight, sex appeal, measurements, and muscle tone) and six non-observable physical attributes (muscular strength, physical coordination, stamina, health, physical fitness, and physical energy level). Participants were then asked to rate the importance of each of these attributes to their physical self-concept on a scale from 1 (*Not at all important*) to 5 (*Very important*). The mean rating for the non-observable attributes was then subtracted from the observable attributes to give a variable in which higher scores reflected greater self-objectification.

#### Statistical Analysis

Initially, correlation analyses were conducted to assess the association between the variables. Following this, confirmatory factor analysis was used to assess whether the expected emotions were separate constructs. Next, a series of ANOVAs were conducted to assess the effect of the manipulation on the expected emotions and anticipated responses. Finally, the indirect effect of the manipulation on the anticipated responses via the expected emotions was assessed using the Process macro (Hayes 2013). The ANOVA and indirect effect analyses were repeated on numerous variables. Therefore, Bonferroni corrections were made to account for the family-wise error rate.

### Results

During the data sorting process it was clear that there were outliers for the age, pride, and anticipated active and self-blame response variables (i.e., scores ±3 standard deviations from the mean). Therefore, logarithmic transformations were performed on pride and the anticipated active and self-blame response variables to correct for these outliers. Moreover, an inverse transformation was performed on the age variable to correct for outliers. A Chi-squared analysis was also performed to make sure that attrition did not vary between conditions (see Zhou and Fishbach 2016). There was not a significant association between condition and attrition, *χ*^*2*^ (1) = .51, *p* = .477, *ϕ* = .05. This suggests that the randomisation in this experiment was not violated by attrition.

#### Associations between Variables

Anticipating an active response was positively associated with expected anger, disgust, shame, inferiority, and rejection (see Table 3). By contrast, pride was negatively associated with anticipating an active response. Similarly, anticipating a passive response was negatively associated with expected anger, disgust, and shame, but positively associated with pride. The anticipation of undertaking a self-blame response was positively associated with expected disgust, shame, inferiority, and rejection, but negatively associated with pride. The anticipation of undertaking a benign response was negatively associated with expecting aversive emotions, but positively associated with pride. Self-objectification was positively associated with anticipated self-blame. Finally, age was negatively associated with expected shame and anticipated self-blame. There were some close associations between the expected emotions. Indeed, expected anger and disgust were closely associated. Moreover, the expected self-critical emotions were closely associated. Therefore, it was important to test whether these were separate constructs.

**Table 3:** Descriptive statistics and correlations between the expected emotions and anticipated responses, Study 2

		Correlations										
	*M* (*SD*)	1	2	3	4	5	6	7	8	9	10	11
Expected emotions												
1. Anger	2.81 (1.28)	–										
2. Disgust	2.97 (1.40)	.84***	–									
3. Shame	2.13 (1.07)	.57***	.64***	–								
4. Inferiority	2.62 (1.19)	.57***	.61***	.73***	–							
5. Rejection	1.88 (.86)	.52***	.53***	.63***	.74***	–						
6. Pride	.22 (.20)	−.51***	−.58***	−.40***	−.43***	−.44***	–					
Anticipated responses												
7. Active response	.33 (.16)	.56***	.50***	.23**	.15*	.15*	−.26***	–				
8. Passive response	2.77 (.93)	−.34***	−.34***	−.16*	−.11	−.08	.19*	−.52***	–			
9. Self-blame response	.23 (.20)	.09	.22**	.45***	.40***	.34***	−.17*	−.08	.13	–		
10. Benign response	1.96 (.71)	−.56***	−.62***	−.40***	−.39***	−.37***	.69***	−.36***	.37***	−.06	–	
Covariates												
11. Self-objectification	−.44 (.95)	−.10	−.08	.02	.01	−.02	.14	−.07	.04	.18*	.05	–
12. Age	1.02 (.01)	.02	−.04	−.15*	−.13	−.03	.05	.13	−.08	−.19*	.01	−.14

*p < .05. **p < .01. ***p < .001

#### Confirmatory Factor Analysis

Confirmatory factor analysis was used to assess whether the six expected emotions were separate constructs. This model was assessed in AMOS (Version 22, Arbuckle 2013) using maximum likelihood estimation. The hypothesised six-factor model fit the data well: χ^2^(89) = 153.57, *p* < .001, CFI = .97, NFI = .93 and RMSEA = .06. This was contrasted against a five-factor model in which expected anger and disgust were combined. This model had adequate fit: χ^2^(94) = 204.98, *p* < .001, CFI = .95, NFI = .91 and RMSEA = .08. However, this model did not fit the data as well as the hypothesised six-factor model: ∆χ^2^(5) = 51.41, *p* < .001. The hypothesised model was also contrasted with a four-factor model in which the expected self-critical emotions (shame, inferiority and rejection) formed a single factor. The fit for this model was adequate: χ^2^(98) = 188.01, *p* < .001, CFI = .96, NFI = .92 and RMSEA = .07. However, the fit of the hypothesised six-factor model was superior: ∆χ^2^(9) = 34.44, *p* < .001. Therefore, based on these analyses, the expected emotions were regarded as six separate constructs. Importantly, further analysis revealed that including the six expected emotions into a regression analysis produced a lowest tolerance value of .23. Because this was greater than .20, it is unlikely that any regression analyses would be bias by multicollinearity (Menard 1995). Therefore, multicollinearity was unlikely to be an issue when conducting the indirect effect analyses.

#### Effect of Sexual Objectification

Next, a series of ANOVAs were conducted to test whether the expected emotions, anticipated responses, and self-objectification varied between the control and objectified conditions. Given that the analyses were being repeated 11 times, Bonferroni corrections were used to control for family-wise error. As such, results with a *p*-value less than .0045 were regarded as significant. Expected anger, disgust, shame and inferiority were higher in the objectified than in the control condition (see Table 4). By contrast, the manipulation did not have a significant effect on expected rejection or pride. Importantly, women anticipated undertaking an active response to a greater extent in the objectified than in the control condition. By contrast, the anticipation of undertaking a benign response was lower in the objectified than in the control condition. Self-objectification and the anticipation of undertaking a passive and self-blame response did not vary between the objectified and control conditions. The analyses were also repeated with self-objectification and age included as covariates. Importantly, including these covariates into the analysis did not alter the results. These results reflect the fact that experiencing overt sexual objectification increased the expectation of feeling some of the negative emotions (i.e., anger, disgust, shame and inferiority) and anticipated active responses, but decreased benign responses.

**Table 4:** The effect of sexual objectification on the expected emotions and anticipated responses, Study 2

	Control *M* (*SD*)	Sexual objectification *M* (*SD*)	*F*(1, 185)	*p*	ηp^2^
Expected emotions					
Anger	2.29 (1.12)	3.30 (1.25)	33.45	<.001	.15
Disgust	2.41 (1.34)	3.50 (1.25)	33.34	<.001	.15
Shame	1.81 (.99)	2.43 (1.06)	17.10	<.001	.09
Inferiority	2.33 (1.18)	2.89 (1.14)	10.75	=.001	.06
Rejection	1.72 (.82)	2.02 (.88)	6.06	.015	.03
Pride	.24 (.21)	.21 (.19)	1.20	.275	.01
Anticipated responses					
Active response	.28 (.15)	.38 (.16)	20.56	<.001	.10
Passive response	2.90 (.92)	2.65 (.93)	3.43	.066	.02
Self-blame response	.22 (.20)	.23 (.19)	.11	.739	<.01
Benign response	2.14 (.70)	1.80 (.69)	10.93	.001	.06
Self-objectification	−.54 (1.02)	−.35 (.87)	1.90	.170	.01

It could be argued that the effect of the manipulation on the expected emotions and anticipated responses may be moderated by self-objectification. Therefore, this was tested using the Process Macro (Model 1, Hayes 2013). In this analysis the independent variable (condition) and the potential moderator (self-objectification) were mean centred. A separate analysis was conducted on each of the expected emotions and anticipated responses. Across all the analyses, the interaction between condition and self-objectification was non-significant (*p*s > .10). These results reflect the fact that self-objectification did not moderate the effect of condition on the expected emotions or the anticipated responses.

#### Indirect Effects

Given that the sexual objectification manipulation had a significant effect on some of the negative expected emotions (see Table 4) and that these emotions predicted the anticipated responses (see Table 3), there was the possibility of an indirect effect of the manipulation on the anticipated responses via the expected emotions (MacKinnon 2008; Preacher and Hayes 2008). Indirect effect analyses were performed using the Process Macro (Model 4, Hayes 2013). In these analyses, the sexual objectification manipulation was the independent variable, the expected emotions were the mediating variables, and the anticipated responses were the dependent variables. The confidence intervals were calculated using 5000 bootstrap resamples. A separate analysis was performed on each of the four anticipated responses. Given that the analysis was repeated four times, 99% confidence intervals were used to ensure that the family-wise error rate did not exceed .05.

##### Anticipated Active Response

**Fig. 1:**
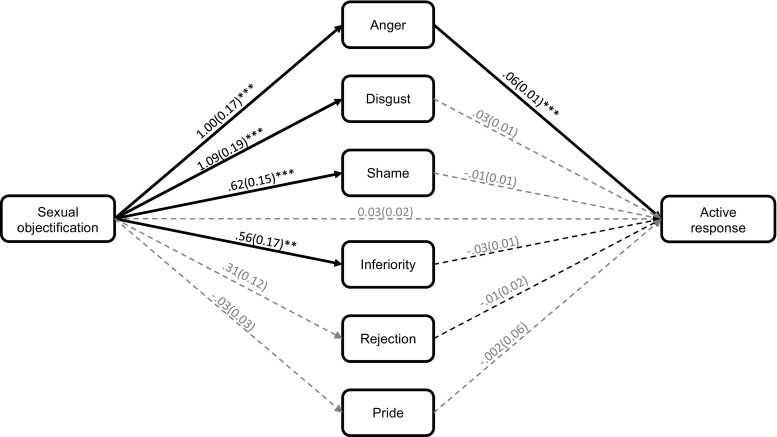
The indirect effect of the sexual objectification manipulation on the anticipation of undertaking an active response via the expected emotions (Study 2). The values represent unstandardised betas and standard errors (in brackets). Pathways were regarded as significant if the *p*-value was below .0125. This was to correct for the family-wise error rate. *p < .0125. **p < .01. *** = *p* < .001

The indirect effect analysis revealed that the significant effect of the sexual objectification manipulation on the anticipation of undertaking an active response (see Table 4) became non-significant after controlling for the expected emotions (see Fig. 1). This analysis also revealed that expected anger positively predicted the anticipation of undertaking an active response. Given that expected anger was predicted by the sexual objectification manipulation and that this emotion uniquely predicted anticipated active responses, there was likely to be an indirect effect of the manipulation via anger. Indeed, the 99% confidence intervals did not contain zero (i.e., were significant) for the indirect effect via expected anger (*B* = .06, *SE* = .02, 99% CI [.02, .13]). By contrast, the confidence intervals contained zero for the indirect effects via expected disgust (*B* = .03, *SE* = .02, 99% CI [−.02, .08]), shame (*B* = −.004, *SE* = .01, 99% CI [−.03, .02]), inferiority (*B* = −.02, *SE* = .01, 99% CI [−.05, .002]), rejection (*B* = −.004, *SE* = .01, 99% CI [−.02, .01]), and pride (*B* = .0001, *SE* = .003, 99% CI [−.01, .01]). These results suggest that sexual objectification had an indirect effect via expected anger. Sexual objectification increased the expectation of feeling anger. In turn, expected anger positively predicted the anticipation of undertaking an active response.

##### Anticipated Passive Response

**Fig. 2:**
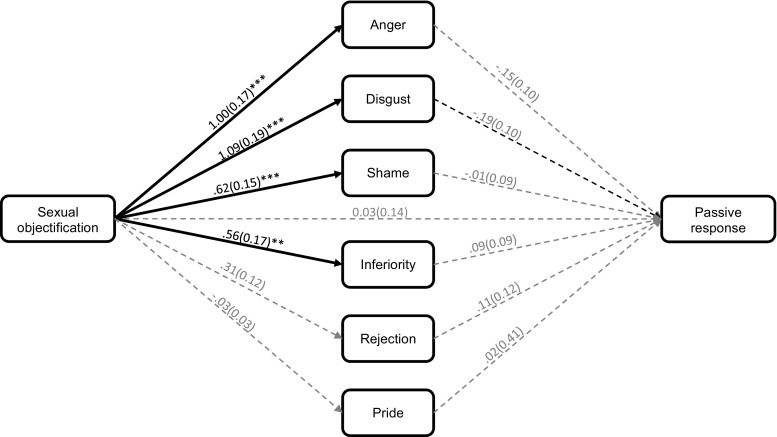
The indirect effect of the sexual objectification manipulation on the anticipation of undertaking a passive response via the expected emotions (Study 2). The values represent unstandardised betas and standard errors (in brackets). Pathways were regarded as significant if the *p*-value was below .0125. This was to correct for the family-wise error rate. *p < .0125. **p < .01. *** = *p* < .001

The indirect effect analysis suggested that after correcting for the family-wise error rate, none of the emotions uniquely predicted anticipating a passive response (see Fig. 2). Similarly, the 99% confidence internals contained zero for the indirect effects via expected anger (*B* = −.15, *SE* = .11, 99% CI [−.48, .11]), disgust (*B* = −.21, *SE* = .12, 99% CI [−.57, .04]), shame (*B* = −.003, *SE* = .06, 99% CI [−.18, .16]), inferiority (*B* = .05, *SE* = .06, 99% CI [−.08, .24]), rejection (*B* = .03, *SE* = .04, 99% CI [−.08, .16]), and pride (*B* = −.001, *SE* = .02, 99% CI [−.07, .06]). These results suggest that being objectified did not increase the anticipation that a passive response via the expected emotions.

##### Anticipated Self-Blame Response

**Fig. 3:**
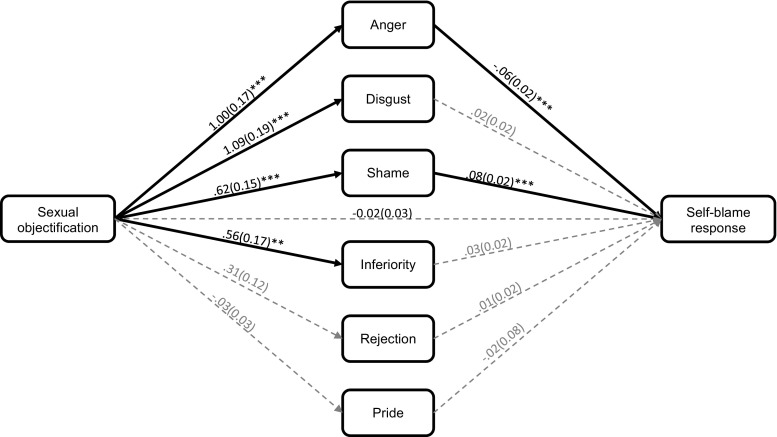
The indirect effect of the sexual objectification manipulation on the anticipation of undertaking a self-blame response via the expected emotions (Study 2). The values represent unstandardised betas and standard errors (in brackets). Pathways were regarded as significant if the p-value was below .0125. This was to correct for the family-wise error rate. *p < .0125. **p < .01. *** = *p* < .001

The indirect effect analysis suggested expected anger and shame were significant predictors of anticipating a self-blame response (see Fig. 3). The combination of this and the significant effect of the manipulation on these variables suggested that there may be an indirect effect. Indeed, the confidence intervals did not contain zero for the indirect effects via expected anger (*B* = −.06, *SE* = .02, 99% CI [−.13, −.01]) and shame (*B* = .05, *SE* = .02, 99% CI [.01, .09]). By contrast, the confidence intervals contained zero for the indirect effects via expected inferiority (*B* = .02, *SE* = .01, 99% CI [−.01, .06]), disgust (*B* = .02, *SE* = .02, 99% CI [−.04, .08]), rejection (*B* = .004, *SE* = .01, 99% CI [−.02, .03]), and pride (*B* = .001, *SE* = .003, 99% CI [−.01, .01]). These findings suggest that expecting to experience shame after sexual objectification is likely to promote, whereas expecting anger, is likely to deter anticipated self-blame.

##### Anticipated Benign Response

**Fig. 4:**
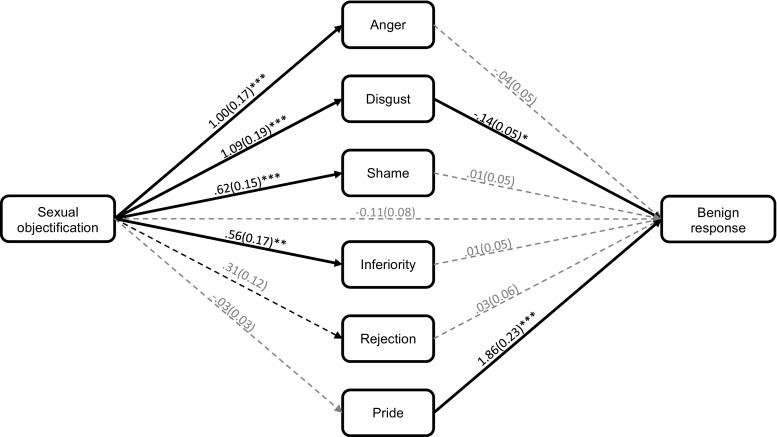
The indirect effect of the sexual objectification manipulation on the anticipation of undertaking a benign response via the expected emotions (Study 2). The values represent unstandardised betas and standard errors (in brackets). Pathways were regarded as significant if the *p*-value was below .0125. This was to correct for the family-wise error rate. *p < .0125. **p < .01. *** = *p* < .001

Expected disgust uniquely predicted the anticipation of a benign response (see Fig. 4). Given that the sexual objectification manipulation has a significant effect on expected disgust, there was a potential indirect pathway from this manipulation to anticipated benign responses via expected disgust. However, after controlling for family-wise error rates (i.e., using 99% confidence intervals), the indirect effect was non-significant (*B* = −.15, *SE* = .07, 99% CI [−.36, .02]). Although pride also uniquely predicted anticipating a benign response, the effect of the sexual objectification manipulation on pride was non-significant, thereby creating a non-significant indirect pathway (*B* = −.06, *SE* = .06, 99% CI [−.21, .08]). Moreover, the confidence intervals contained zero for the indirect effects via expected anger (*B* = −.04, *SE* = .06, 99% CI [−.20, .11]), shame (*B* = .004, *SE* = .03, 99% CI [−.09, .10]), inferiority (*B* = .004, *SE* = .03, 99% CI [−.09, .11]), and rejection (*B* = .01, *SE* = .02, 99% CI [−.04, .06]). This suggests that objectification did not increase the anticipation of a benign response via the expected emotions.

##### Covariates

The above indirect effect analyses were repeated with self-objectification and age entered into the models as covariates. The significant indirect effects outlined previously remained significant after controlling for these covariates. This suggests that the indirect effects were not due to age or self-objectification.

### Discussion

This study supported Study 1 by demonstrating the role of expected emotions in predicting anticipated responses to sexual objectification. Indeed, Study 2 supported Study 1 and the hypotheses in demonstrating that expected anger positively predicts anticipated active responses, expected disgust negatively predicts anticipated benign responses, and that expected shame positively predicts anticipated self-blame. Importantly, Study 2 enhanced the previous study by demonstrating the role of pride in promoting the anticipation of benign responses. Furthermore, the use of an experimental design and indirect effect analysis allowed for stronger causal inferences to be made. Indeed, the indirect effect analyses suggested that there is likely to be an indirect effect of sexual objectification on some of the anticipated responses via the expected emotions. Therefore, the present findings support the idea that the emotions that are expected to be experienced following sexual objectification influence the type of response that women believe they would undertake.

Expected disgust was the only aversive emotion to negatively predict anticipated benign responses in both studies. This is likely to reflect the role of disgust in guiding moral judgements. Disgust increases moral condemnation for violations of purity (Horberg et al. 2009). Therefore, targets of purity violations (e.g., sexual objectifying behaviours) may be particularly likely to condemn the action when they feel disgust. This may have resulted in women who felt disgust towards the sexually objectifying behaviour being unlikely to anticipate a benign response.

Expected inferiority and rejection did not significantly predict the anticipated responses in either of the studies. This may be partly due to the fact that these variables were closely associated with shame. Moreover, it may also be the case that these expected emotions may predict other anticipated responses that were not assessed in this research. For example, rejection is associated with withdrawal (Gausel and Leach 2011). Although there is some overlap between withdrawal and passive responses, they are distinct constructs. Indeed, withdrawal involves actively avoiding a situation (i.e., not going back to the gym), whereas passive responses involves inaction (i.e., pretending the action did not happen). Therefore, I may have been more likely to find that expected rejection predicted behaviour if I measured anticipated withdrawal responses.

Interestingly, there were some differences between the results of Studies 1 and 2. First, expected anger predicted the anticipation of passive and benign responses in Study 1 but not Study 2. Second, expected disgust predicted anticipated active and passive responses in Study 1 but not Study 2. These differences are likely to reflect the association between expected anger and disgust. There was a stronger correlation between expected anger and disgust in Study 2 than in Study 1. As a result, both of these expected emotions may have been less likely to uniquely predict the anticipated responses in the latter study. Given that both are emotions based in moral outrage and given that they are strongly correlated, it could be argued that these expected emotions should be combined. Indeed, some previous research has combined these closely associated emotions (Bastian et al. 2013). Although further analyses suggested that combining these expected emotions increased the consistency across the two studies, my confirmatory factor analysis suggested that these were distinct constructs. Moreover, including the separate expected anger and disgust constructs into a regression analysis did not cause multicollinearity. Because of this, I regarded these expected emotions as separate constructs.

## General Discussion

Previous research has identified numerous responses that may be undertaken following instances of sexual objectification. However, to date there has been little research assessing the factors that promote the different types of responses. The present studies demonstrate the role of expected emotions in promoting and deterring different anticipated responses to sexual objectification. Expected anger led women to anticipate they would undertake active responses, and expected disgust was negatively associated with the belief that benign responses would be undertaken. Expected shame increased the belief that self-blame would be undertaken, and expected pride was positively associated with the anticipation of undertaking benign responses. Therefore, my research enhances previous literature (e.g., Chaudoir and Quinn 2010; Fairchild and Rudman 2008) by demonstrating the importance of different expected emotions in influencing the anticipated responses following instances of sexual objectification.

These findings were strengthened through the design of the studies. The use of experimental methods in Study 2 allowed for a causal direction to be inferred. As such, it can be concluded that sexual-objectification promotes the anticipated responses via the expected emotions. Moreover, a series of covariates was assessed across the two studies. Study 1 tested whether the relationships remained after controlling for body surveillance and body shame, whereas Study 2 tested whether relationships remained after controlling for self-objectification. Importantly, the expected emotions predicted the anticipated responses after controlling for these covariates. These findings demonstrate that the expected emotions were robust predictors of the anticipated responses, thereby further strengthening these results.

### Limitations and Future Research Directions

Although these findings are informative, it is also important to consider the limitations of the present research. There may be discrepancies in the findings of studies conducted in-person and using vignettes (e.g., Woodzicka and LaFrance 2001). By asking women to rate how they thought they would respond, my research assessed anticipated responses rather than actual responses. Given the discrepancy between anticipated and actual outcomes (Wilson and Gilbert 2005) and given the gap between the willingness to act and behaviour (Sheeran 2002), it is important for future research to assess the role of actual emotions in promoting and deterring actual responses to sexual objectification.

There are also a number of other limitations. First, this research focused on the expected emotions toward and anticipated responses to body evaluation (i.e., receiving an inappropriate sexual comment). It is important to extent these findings to other types of sexual objectification. For example, it is important to test whether similar processes occur when women receive unwanted explicit sexual advances. Second, although the expected emotions and anticipated responses that were assessed in this research were based on previous studies, there may be other emotions and responses elicited in such situations. Therefore, future studies should use open-ended questions to determine the different emotions and responses that may be elicited. Third, a substantial proportion of participants dropped out of the study after giving consent. This is common in online studies (Birnbaum 2004). Although there was no evidence of condition-dependent attrition (see Zhou and Fishbach 2016), the drop-out rate may have introduced other forms of sampling bias. However, the use of an online study was likely to recruit a more diverse sample than other types of studies, such as those that use student samples. Therefore, the sample was likely to be more diverse than if a laboratory study was used. Finally, in the vignettes the actions occurred in a semi-public location. It is also important to assess whether the effects are applicable to sexual objectification that occurs in other locations, such as street harassment. Addressing these limitations would further advance this line of research.

The present research provides strong evidence for the fact that expected emotions predict the type of response that is believed would be undertaken following instances of sexual objectification. Given this, it is also important to determine the factors that are likely to predict the emotions that are elicited in such situations. For example, endorsing sexist views may undermine perceptions of inequality and the willingness to undertake confrontational action (Becker and Wright 2011; Glick and Fiske 2001). Therefore, these factors may undermine feelings of anger following instances of sexual objectification and thus reduce active responses. However, future research is needed to assess the extent to which these and other factors may influence the type of emotion that is elicited following instances of sexual objectification.

### Practice Implications

Despite these limitations, it is also important to discuss the practical implications of this research. Research has suggested that self-blame and benign responses are positively related to self-objectification (Fairchild and Rudman 2008) and that self-objectification may be harmful to well-being (Fredrickson and Roberts 1997; Noll and Fredrickson 1998). Therefore, it is important to reduce the likelihood of self-blame and benign responses following sexual objectification. My research demonstrated that these anticipated responses were likely when shame or pride are expected to be experienced. These emotions are likely to be felt when an action has been attributed to the self (Smith and Lazarus 1993). This suggests these emotions, and their harmful responses, may be avoided when external attributes are made following instances of sexual objectification, such as blaming the perpetrator. Based on this reasoning, it is important to develop campaigns and educational materials that demonstrate the harm caused by objectification and emphasise the fact that the blame should be with the perpetrator rather than the target. Such campaigns and educational materials should decrease the likelihood of shame and pride being experienced. These should, in turn, reduce self-blame and benign responses.

This is not to say that the emphasis for tackling sexual objectification should be placed on the target. Indeed, it is also important to target the perpetrators of such actions. Research has suggested that men may feel badly when considering the negative treatment of women and that this may motivate them to make amends (Branscombe 1998; Schmitt et al. 2008). Therefore, campaigns and educational materials that target the perpetrator and emphasise the harm of objectification may also reduce the likelihood of men engaging in objectifying behaviours.

### Conclusion

The present research tested the role of expected emotions in promoting and deterring different anticipated responses to sexual objectification. It was hypothesised that expecting to feel emotions based in moral outrage (i.e., anger and disgust) would lead women to believe they would undertake active responses and reduce passive responses, that expecting self-critical emotions (i.e., shame and inferiority) would promote anticipated self-blame, and that expecting pride would increase the anticipation of benign responses. The majority of these hypotheses was supported across my two studies. Therefore, my research suggests that it is important to consider the role of emotions in influencing women’s anticipated responses to sexual objectification.

## Electronic Supplementary Material

ESM 1 (DOCX 27 kb)
