# Supplementary material for: Responding to Sexual Objectification: The Role of Emotions in Influencing Willingness to Undertake Different Types of Action
Source: Sex Roles. 2018 Apr 9;80(1):25–40. doi: 10.1007/s11199-018-0912-x (PMC6318258; doi:10.1007/s11199-018-0912-x)
Supplement: Supplementary file 1 — (DOCX 27 kb) [file 11199_2018_912_MOESM1_ESM.docx]

Online supplement for Shepherd, L. (2018). Responding to sexual objectification: The role of emotions in influencing willingness to undertake different types of action. *Sex Roles*. Lee Shepherd, Northumbria University. Email: [lee.shepherd@northumbria.ac.uk](mailto:lee.shepherd@northumbria.ac.uk)

**Vignettes**

In Study 1, the following vignette was used:

*Please imagine that you are exercising at your local gym. After finishing on the treadmill you decide to have a short break to get some water. As you are having this break a man that you have never met before comes up to you and starts a conversation. For a few minutes you make polite conversation. This man then says to you: “You were looking good on the treadmill. It seems to be working for you. You have a great body and an amazing ass.”*

In Study 2, participants in the control condition received the following vignette:

*Please imagine that you are exercising at your local gym. After finishing on the treadmill you decide to have a short break to get some water. As you are having this break a man that you have never met before comes up to you and starts a conversation. For a few minutes you make polite conversation. This man then says to you: “You were looking good on the treadmill. Your exercise regime seems to be working for you.”*

Participants in the sexual objectification condition received the following vignette:

*Please imagine that you are exercising at your local gym. After finishing on the treadmill you decide to have a short break to get some water. As you are having this break a man that you have never met before comes up to you and starts a conversation. For a few minutes you make polite conversation. This man then says to you: “You were looking good on the treadmill. Your exercise regime seems to be working for you. You have a great body and an amazing ass.”*

**Anticipated Response Scales**

Fairchild and Rudman (2008) asked participants to rate how they typically responded to sexually objectifying behaviours. However, given I asked women to imagine a situation, the wording of the items had to be altered. The exact wording for each item is below. This scale was used in both studies. Each item was rated on a five-point scale that contained the following labels: Not at all likely, Slightly likely, Somewhat likely, Very Likely, and Extremely likely.

**Anticipated passive response subscale**

How likely are you to ‘‘blow it off’’ and act like you do not care?

How likely are you to let it go?

How likely are you to ignore the whole thing?

How likely are you to not do anything?

How likely are you to act like you did not notice?

How likely are you to forget the whole thing?

How likely are you to pretend that nothing has happened?

**Anticipated self-blame response subscale**

How likely are you to think that you had probably brought it on yourself?

How likely are you to blame yourself for what happened?

How likely are you to think he probably would not do it if you had dressed differently?

How likely are you to feel stupid for letting yourself get into the situation?

**Anticipated benign response subscale**

How likely are you to consider it flattering?

How likely are you to assume he means well?

How likely are you to figure he must really like you?

How likely are you to assume he was trying to be funny?

How likely are you to treat it as a joke?

**Anticipated active response subscale**

How likely are you to let him know you do not like what he is doing?

How likely are you to let him know how you feel about what he is doing?

How likely are you to talk to someone about what happened?

How likely are you to report him?
